# Social Class and Personality: The Effects of Educational Mobility on Personality Trait Change

**DOI:** 10.1177/19485506251326333

**Published:** 2025-03-31

**Authors:** Anatolia Batruch, Manon A. van Scheppingen

**Affiliations:** 1 Université de Lausanne, Switzerland; 2 Tilburg University, The Netherlands

**Keywords:** Big Five, personality change, social mobility, social class, education

## Abstract

Transitioning into young adulthood often brings about significant changes in personality traits. However, the reasons behind these personality changes remain unclear. This study integrates insights from research on personality development and the psychology of social class to study how the construction of one’s social class identity in young adulthood might trigger changes in personality traits (i.e., Big Five, locus of control, and risk-taking). We tested our preregistered hypotheses in the context of educational mobility, using data from the German Socio-Economic Panel (SOEP) study (*N* = 4,776). Specifically, we investigated personality changes of young adults whose parents did not go to university, comparing those who are educationally mobile (i.e., go to university) with those who do not during the study period. Overall, the results indicated that upward educational mobility only leads to changes in risk-taking. Theoretical implications for the psychology of class mobility (selection vs. socialization effects) are discussed.

Personality traits refer to stable differences in how individuals think, feel, and behave. A large body of research has shown that personality traits can change over time, with young adulthood being a particularly important period of change (see Bleidorn et al., 2022; Roberts et al., 2006; Robins et al., 2001; Schwaba & Bleidorn, 2018; Specht et al., 2011; Wright & Jackson, 2023). This is unsurprising, given that entering adulthood involves navigating numerous significant life transitions in both personal (e.g., first romantic relationships, leaving the parental home, developing new friendships, or adopting different identities and perspectives; Bleidorn et al., 2018) and professional domains (e.g., graduating from high school; Bühler et al., 2023). It also coincides with the construction of one’s future social class. Together, these experiences may affect personality to the extent that the post-high-school years are associated with additional gains in openness (*d* = 0.20), agreeableness (*d* = 0.32), and conscientiousness (*d* = 0.32), and decreases in neuroticism (*d* = −0.27; Lüdtke et al., 2011). For first-generation students (whose parents did not go to university), these social experiences additionally entail adapting to a new social class environment. In this study, we contribute to the literature by using a case–control design to examine how experiencing educational mobility impacts changes in personality traits (i.e., the Big Five, locus of control, and risk-taking) that predict key life outcomes (e.g., health, life satisfaction, professional success; Dohmen et al., 2011; Ryon & Gleason, 2014; Soto, 2021).

## Social Class and the Transition to University

A wide array of research in the psychology of social class literature postulates that social class—usually measured with one or a combination of the following indicators: educational attainment, income, and subjective status—is a cultural environment that exerts strong socializing pressures on individuals. Repeated experiences in specific social class cultures should lead individuals to endorse different behavioral norms, values, and perspectives (Batruch et al., 2025; Martin & Côté, 2019, for a review, see Manstead et al., 2018). While this literature does not directly examine its impact on personality, findings in personality psychology show a correlation between social class and personality traits (Ayoub et al., 2018; Hughes et al., 2021). For instance, children from high socio-economic status (SES) homes, as compared to low-SES homes have higher levels of conscientiousness, extraversion, and openness and lower levels of neuroticism (Jonassaint et al., 2011). In adults, positive associations are also found between SES and extraversion, openness, emotional stability, and conscientiousness (Chapman, 2009; Lahey, 2009).

Most young adults will remain in a similar social class environment to that of their parents. However, for a significant minority of adults who are experiencing upward social mobility (e.g., 20.4% in Germany, 24.3% in the United States), transitioning to adulthood also requires adapting to a new cultural environment as they enter university (Balestra & Tonkin, 2018). During this period, first-generation students report experiencing a wide range of identity-related challenges. Upon arrival at university, when compared to continuing-generation students (individuals with at least one parent who has attended university; Jury et al., 2017), they experience difficulty embracing their new identity as college students, which they perceive as incompatible with their previous identity (Iyer et al., 2009; Matschke, 2022; Reay et al., 2009), and report feeling that they “do not belong” in the college context (Harackiewicz et al., 2014; Ostrove & Long, 2007; Pittman & Richmond, 2007; Rubin, 2012; Soria et al., 2013).

Entering university is a major life transition for all students as they learn to behave like a highly educated person, but for first-generation students, these adaptations may be less intuitive. This new social role presents status challenges (e.g., learning new technical skills, learning to behave like a leader, confidently presenting original ideas in class) as well as belongingness challenges (e.g., learning to connect with people from a different social group, having to adapt to new behavioral norms; Roberts & Nickel, 2021). Both challenges entail embodying behaviors and adapting to norms that are not widespread in less-educated social class environments (Kraus & Stephens, 2012).

## Social Class Change and Personality

According to models describing bottom-up mechanisms for personality change (e.g., the neo-socioanalytic theory or the TESSERA model), embodying behaviors and adapting to norms are processes that could trigger changes in first-generation students toward resembling more their higher social class counterparts (Quintus et al., 2021; Wrzus & Roberts, 2017). Indeed, individuals may not necessarily change as a result of a conscious decision to alter some aspects of their personality. More often, changes might occur because an individual is confronted with new environments or social roles, which elicit expectations and a desire to “fit in” (Jackson & Wright, 2024). Repeated exposure to these new situations or cultural environments may lead to changes in thoughts, feelings, and behaviors, which over time become more habitual and eventually internalized as part of their self-concept (Roberts & Nickel, 2021; Wrzus & Roberts, 2017).

Previous research highlights some broader effects of cultural shifts on personality change (for a review, see Lu et al., 2023). Multicultural experiences, such as living abroad, intercultural relationships, or multilingualism, have been shown to influence individuals by exposing them to diverse cultural frameworks, including varying knowledge, beliefs, values, norms, and practices (Adam et al., 2018; Lu, 2022; Maddux et al., 2021). Over time, individuals’ personality adapt, to some extent, to align with their specific cultural environments. Longitudinal research has demonstrated that East Asians engaging with American culture develop higher self-esteem, which is generally more prevalent among Americans (Heine et al., 1999). Similarly, a comparison of first-generation Japanese immigrants in the United States with Japanese and American monoculturals revealed that immigrants’ personality became more “American” and less “Japanese” through the process of acculturation (Güngör et al., 2013). The degree of personality change correlates positively with exposure to a new culture, (e.g., length of residence, McCrae et al., 1998). Even within-country changes in culture can shape personality: Roberts and Helson (1997) found that increases in individualism in the United States from 1950 to 1985 were accompanied by increases in narcissism and decreases in norm adherence in women.

Building on this body of research, this study examines personality changes in first-generation university students, a group undergoing both a significant transition into a new social role associated with a new social class, and exposure to peers from that social class. Effects of life events on mean-level personality change tend to be small, yet their impact can vary significantly across individuals (Bleidorn et al., 2021). Compared to other young adults, changes over time might be stronger in first-generation students, who are exposed to a particularly novel environment where they are expected to behave more like members of a higher social class.

To the best of our knowledge, only one longitudinal study has examined differences in personality trait change across SES groups during students’ transition to university. The study focused on a group of 575 adolescents in Australia who were followed for a maximum of 8 years, of whom 33% entered university within this timeframe (Kassenboehmer et al., 2018). This study showed that increases in agreeableness during this transition were especially pronounced among students from low-SES backgrounds (i.e., measured only with the father’s occupational prestige score). No other differences in Big Five personality change were found.

The design of our study significantly improves upon Kassenboehmer and colleagues’ (2018) study in three key ways. Most importantly, by using propensity score matching, we compared first-generation students to a control group who were similar in their pre-event personality trait levels, parental educational level, parental occupational prestige, parental income, and other background factors. This matched control group is crucial because personality traits and other background variables are found to be predictors of educational attainment (Damian et al., 2015; Ou & Reynolds, 2008). Without accounting for these selection effects, it remains unclear whether differences in personality change are caused by the transition itself or by pre-existing group differences. We also draw on a large representative sample and consider *both* parents’ educational levels to identify first-generation students. Thus, we aim to provide a truly robust test of whether the transition to university induces personality trait changes in first-generation students.

## Hypotheses

We draw from theory and research on personality psychology and the psychology of social class to formulate hypotheses about how the cultural changes associated with the transition to university may translate into personality change in young adults. Our main hypotheses (preregistered at: https://osf.io/mcrpn and https://osf.io/ztc7y) are that young adults from lower-SES families who attend university as compared to those who do not, present more positive changes in extraversion, conscientiousness, openness to experience, emotional stability, locus of control, and risk-taking propensity. Because theory and previous research are inconclusive regarding whether and how agreeableness might be affected by this transition, we did not formulate a specific hypothesis for this trait.

To test our hypotheses, we focused on comparing the personality trait development of young adults experiencing educational mobility with those who do not. We measured SES using the most common indicator in educational psychology: first-generation versus continuing-generation students (Stephens et al., 2012). We followed a representative sample of young adults from before the transition to university (i.e., at age 17) up to 10 years later. We controlled for pre-existing differences in personal characteristics *and* parental background variables by using propensity score matching. Thus, we provide a strong test of whether educational mobility triggers personality trait change.

## Method

### Sample

Data were drawn from the German Socio-Economic Panel study (SOEP, 2022; version 37, 1984–2020), produced by the Deutsches Institut für Wirtschaftsforschung (DIW) in Berlin. The SOEP is a large, nationally representative, longitudinal study of German households. From 2000 onward, the Big Five traits, locus of control, and risk-taking propensity were measured at age 17 in the annual youth questionnaire. Once participants transitioned to the adult questionnaire at age 18, the Big Five traits were measured in 2005, 2009, 2012/2013, 2017, and 2019; locus of control in 2005, 2010, 2015/2016, and 2020; and risk-taking propensity in 2004, 2006, and from 2008 to 2020.

Since these traits were measured in different years, samples were selected separately but followed the same inclusion criteria. Combining data from the youth and adult questionnaires, we selected respondents who were under 20 years old (i.e., the age at which most German students have started university) at the first personality measurement, and who completed the Big Five personality, locus of control, or risk-taking items at least twice. Only respondents with available data on their own and their parents’ education—derived from SOEP-generated variables merging all household information—were included (see full documentation: https://www.diw.de/documents/publikationen/73/diw_01.c.600609.de/diw_ssp0537.pdf).

This selection resulted in a sample of 4,776 individuals. For our main analysis, we identified two subsamples. The *upward mobility* sample included individuals who entered higher tertiary education between their first and last personality measurement, with parents lacking tertiary degrees (i.e., the treatment sample; *n* = 345 for Big Five traits, *n* = 310 for locus of control, and *n* = 429 for risk-taking propensity). The *stable low* sample included individuals who did not enter tertiary education and whose parents did not hold a tertiary education degree (i.e., the control sample; *n* = 1,866 for Big Five traits, *n* = 1,715 for locus of control, and *n* = 2,519 for risk-taking propensity). Tables 1–3 show responses per subsample and time point after propensity score matching. In the upward mobility sample, the mean recorded duration in tertiary education was 2.97 years (*SD* = 2.38). At the last observation, 27.6% of the upward mobility sample had obtained their tertiary degree, and 30.1% had entered the labor market. Of the stable low sample, 46.4% entered the labor market during the observation period.

**Table 1:** Sample Sizes and Average T-Scores for the Big Five Personality Traits Across Measurement Waves

Measurement wave	Statistic	Upward mobility sample								Matched stable low sample						
		Age (years)	Distance to transition (years)	EX	A	C	ES	O	*n*	Age (years)	EX	A	C	ES	O	*n*
T1	*M*	17.18	−2.10	50.04	50.27	50.83	50.57	50.22	345	17.15	49.46	50.3	50.39	50.59	49.84	551
	*SD*	0.51	0.48	9.21	9.61	9.82	10.12	8.87		0.48	10.24	9.70	9.64	9.31	9.41	
T2	*M*	20.23	0.95	49.82	49.45	52.77	50.35	50.10	345	19.96	50.62	50.50	53.44	50.77	49.56	551
	*SD*	1.51	1.63	9.11	9.78	9.50	10.50	9.35		1.63	9.43	9.83	8.77	10.01	9.99	
T3	*M*	23.65	4.37	50.04	49.02	54.75	51.18	50.32	294	23.30	50.36	49.62	54.82	51.70	49.69	379
	*SD*	1.75	1.96	9.59	10.6	8.60	10.54	9.41		1.96	8.75	9.35	7.86	10.08	9.61	
T4	*M*	27.17	7.89	49.41	49.22	55.24	51.82	49.13	118	27.02	49.84	49.86	55.65	49.81	48.72	142
	*SD*	2.09	2.25	10.43	10.52	8.52	11.27	10.01		2.25	9.97	9.39	7.84	10.35	9.98	
T5	*M*	30.23	10.41	50.66	48.72	56.11	51.77	51.44	52	30.04	50.09	49.03	55.80	51.26	48.96	62
	*SD*	1.44	1.55	10.04	10.61	8.44	11.96	9.61		1.55	9.72	11.12	7.67	11.38	9.77	

*Note.* EX = extraversion; A = agreeableness; C = conscientiousness; ES = emotional stability; O = openness. Reported T-scores correspond with the non-parametric estimates in Figure 2. Samples are matched on background variables and personality traits measured at T1. For an overview of all matching variables, see Tables S1–S3.

**Table 2:** Sample Sizes and Average T-Scores for the Locus of Control Across Measurement Waves

Measurement wave	Statistic	Upward mobility sample				Matched stable low sample		
		Age (years)	Distance to transition (years)	Locus of control	*n*	Age (years)	Locus of control	*n*
T1	*M*	17.18	−1.96	50.37	310	17.16	50.84	489
	*SD*	0.50	1.64	8.28		0.49	8.81	
T2	*M*	20.76	1.61	49.45	310	20.68	48.51	489
	*SD*	1.72	2.24	8.67		1.92	8.35	
T3	*M*	25.76	6.61	49.36	216	25.68	49.21	250
	*SD*	1.72	2.24	7.98		1.92	8.33	
T4	*M*	30.76	11.61	50.18	67	30.68	48.41	73
	*SD*	1.72	2.24	7.62		1.92	7.04	

*Note.* Reported T-scores correspond with the non-parametric estimates in Figure 2. Samples are matched on background variables and personality traits measured at T1. For an overview of all matching variables, see Tables S1–S3.

**Table 3:** Sample Sizes and Average T-Scores for Risk-Taking Across the Measurement Waves

	Upward mobility sample				Matched stable low sample			
Measurement wave	Statistic	Age (years)	Distance to transition (years)	*n*	Risk-taking	Age (years)	Risk-taking	*n*
T1	*M*	17.26	−2.08	49.30	429	17.21	49.25	718
	*SD*	0.54	1.73	8.88		0.50	9.30	
T2	*M*	18.72	−0.63	47.86	429	18.64	48.74	718
	*SD*	0.94	1.79	8.80		0.97	9.61	
T3	*M*	19.98	0.63	47.11	423	19.97	48.11	666
	*SD*	1.20	1.88	9.54		1.32	9.69	
T4	*M*	21.05	1.70	46.62	411	21.07	48.94	581
	*SD*	1.26	1.93	9.62		1.42	9.30	
T5	*M*	22.08	2.74	47.09	379	22.16	49.33	494
	*SD*	1.29	1.96	8.97		1.49	9.45	
T6	*M*	23.14	3.79	47.07	333	23.23	48.76	406
	*SD*	1.32	1.99	9.07		1.52	9.68	
T7	*M*	24.18	4.84	47.02	273	24.27	48.28	317
	*SD*	1.36	2.02	9.55		1.56	9.79	
T8	*M*	25.19	5.84	46.57	219	25.30	49.14	254
	*SD*	1.37	2.03	9.83		1.58	9.05	
T9	*M*	26.25	6.91	46.77	154	26.35	48.84	187
	*SD*	1.38	2.04	9.09		1.63	9.85	
T10	*M*	27.25	7.88	45.87	110	27.38	47.53	135
	*SD*	1.36	2.00	8.61		1.70	9.17	
T11	*M*	28.32	8.95	45.87	89	28.47	47.24	102
	*SD*	1.52	2.11	8.53		1.85	8.50	
T12	*M*	29.36	9.99	44.61	77	29.50	45.80	89
	*SD*	1.57	2.13	9.38		1.88	9.56	
T13	*M*	30.39	11.01	45.39	59	30.51	49.13	72
	*SD*	1.53	2.10	9.81		1.85	8.69	

*Note.* Reported T-scores correspond with the non-parametric estimates in Figure 2. Samples are matched on background variables and personality traits measured at T1. For an overview of all matching variables, see Tables S1–S3.

We also selected a *stable high* sample, which consisted of individuals who entered tertiary education between their first and last personality measurement, and whose parents held a tertiary education degree (*n* = 455 for Big Five traits, *n* = 427 for locus of control, and *n* = 423 for risk-taking propensity).

This sample was used to explore whether personality changes were unique for the upward mobility sample or common to all individuals transitioning to university.

### Measures

#### Big Five

The Big Five personality traits were measured using a 15-item version of the Big Five Inventory (BFI-S) on a scale from 1 (does not apply) to 7 (applies fully), either through a self-administered questionnaire, a face-to-face interview, or a telephone interview. This version was derived from the longer 44-item version, by selecting three items per trait that maximized coverage of the trait (John et al., 1991; Lang et al., 2011). The low number of items per trait is reflected in the relatively low Cronbach’s alphas across measurement occasions: extraversion (α = .66–.74), agreeableness (α = .42–.51), conscientiousness, (α = .58–.67). emotional stability, (α = .57–.66), and openness (α = .56–.59). The BFI-S has shown acceptable convergent validity, discriminant validity, and test–retest stability (Gerlitz & Schupp, 2005; Hahn et al., 2012; Lang et al., 2011).

#### Locus of Control

The locus of control scale assesses the extent to which individuals believe they can (or cannot) determine events in their own lives (Nolte et al., 1997). Participants are presented with statements that relate to different attitudes toward life and the future and asked to rate their level of agreement with the statements on a scale of 1 (not at all) to 7 (absolutely). We followed the recommendation of the SOEP Scales Manual by aggregating seven (items 1, 2, 3, 5, 7, 8, and 10) of the 10 items to improve scale reliability (Richter et al., 2017). Across the four measurement occasions, Cronbach’s alpha ranged from between .50 and .70.

#### Risk-Taking

General risk-taking propensity was assessed with the individual item: “Are you generally a person who is fully prepared to take risks or do you try to avoid taking risks?” using a scale from 0 (risk averse) to 10 (fully prepared to take risks). The test–retest correlation pooled across three waves (2005, 2006, and 2009) was .60 (Richter et al., 2017).

We transformed the Big Five traits, locus of control, and risk-taking propensity into T-scores using the grand mean and standard deviation of the total sample of respondents that participated in the youth questionnaire at age 17 (*N* = 9,370). T-scores are standardized scores with a mean of 50 and a standard deviation of 10. Effect sizes of 2 are considered small, 5 medium, and 8 large (Cohen, 1988).

#### Education Level

Education level was measured using two categories: (a) vocational education or lower and (b) higher education. The “vocational education or lower” category comprises primary school, secondary school, and educational tracks that lead to a skilled (blue-collar) profession. In the higher education category, we included educational tracks recognized by the state as tertiary education (i.e., university, university of applied sciences, special technical school, civil service training, and technical engineering school). For respondents’ education level, we used the maximum education level ever attended between the first and last personality measurements. For parental education level, we used the maximum education level ever completed.

### Analyses

We used maximum likelihood estimation with robust standard errors (MLR) to handle missing outcome data in latent growth curve models. We used Bayesian Stochastic Regression Imputation to address missing data, as missing values are not allowed when using propensity score matching (Coffman et al., 2020; Stuart, 2010). For each outcome measure—Big Five traits, locus of control, and risk-taking, we propensity-score matched (Rosenbaum & Rubin, 1983) the upward mobility sample to the stable low (control) sample on numerous covariates. Matching with replacement minimized differences in propensity scores and ensured robust comparisons (Dehejia, 2002). Each upward mobility participant was matched with the three closest controls based on propensity scores, using a caliper width of 0.2 *SD* of the logit for close matches (Austin, 2011). Details on the matching procedure are provided in the Supplemental Material.

Matching was performed in two steps. First, we matched on background variables (excluding personality traits) to test whether the upward mobility and control samples differed in personality at the initial time point (T1), assessed before entering tertiary education. Matching variables included parental (e.g., job prestige), personal (e.g., age), and survey-related (e.g., survey year) factors likely to influence personality differences (see Tables S1–S3). We matched at T1 instead of the transition point to ensure more complete, unbiased data on personality traits, and pre-event variables. For most participants (>80%), T1 corresponds to age 17, when respondents were in secondary education, minimizing transition-related confounds (e.g., relocation, meeting fellow students). Logistic regression tested whether T1 personality traits predicted upward mobility. We used logistic regression to examine whether personality at T1 was associated with upward mobility in the following years.

Second, we added personality traits at T1 as matching variables, to control for pre-existing personality differences that can predict moving up in educational level (i.e., selection effects). The matched samples from this step were used to analyze change patterns in the upward mobility and stable low samples. Tables S1–S3 show standardized differences for all variables before and after matching. Propensity score matching reduced standardized differences between groups to below .10. R code for analyses is available at https://osf.io/mkqut/.

To examine personality changes, we used latent growth curve modeling in Mplus (Muthén & Muthén, 1998–2017), employing T-scores to account for individually varying observation times between participants. For the upward mobility sample, T-scores were centered at the year before entering higher education, allowing us to assess the university environment’s influence on personality. For the stable low sample, we created a timeline centered at age 19 (the average age one year before higher education entry), ensuring comparability.

For each personality trait and sample, we identified the best-fitting model—no change (intercept-only), linear, or quadratic—using the Bayesian Information Criterion (BIC), where lower values indicate a better fit (Schwarz, 1978). After establishing the best model for each subsample, we used multiple-group latent growth curve models to compare personality development between the upward mobility and stable low samples.

In addition to these main analyses, we explored differences in personality change between the upward mobility and stable high samples using multiple-group latent growth curve models. Notably, we were unable to adequately match these groups on background variables due to inherent differences in parental educational levels.

### Transparency and Openness

Analyses were performed using Mplus version 8 (Muthén & Muthén, 1998–2017) and R version 4.1.3 (R Core Team, 2021), packages *haven* (Wickham & Miller, 2020) and *Matchit* (Ho et al., 2011). All code to prepare the data and execute the final models is available at the following Open Science Framework repository: https://osf.io/mkqut/. The preregistration of our hypotheses and analyses plan for the Big Five traits and locus of control is available at: https://osf.io/mcrpn. We later added an addendum to preregister our hypotheses and analyses plan for risk-taking propensity: https://osf.io/ztc7y. Previous studies using the same dataset (SOEP) can be found at: https://www.diw.de/en/diw_01.c.789503.en/publications_based_on_soep_data__soeplit.html. Access to the SOEP data can be requested at: https://www.diw.de/en/diw_01.c.601584.en/data_access.html.

## Results

### Differences in Personality Before Entering Higher Education

**Figure 1:**
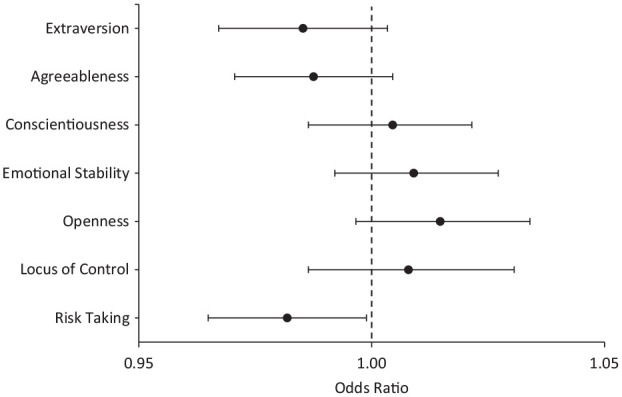
Odds Ratios and 95% Confidence Intervals of Logistic Regression Analyses Predicting Upward Educational Mobility With Personality Traits After Propensity Score Matching *Note.* Propensity score matching was done on background variables but not on personality traits. For an overview of all matching variables, see Tables S1–S3.

Table S4 and Figure 1 show the results of logistic regression analyses that predict upward educational mobility before and after propensity score matching. Before matching, high openness, a more internal locus of control, and low risk-taking were predictors of upward educational mobility. Agreeableness, extraversion, and conscientiousness did not significantly predict upward educational mobility. After matching on parental and participants’ background characteristics, the effects of openness and locus of control became insignificant, leaving only low risk-taking as a significant predictor of upward educational mobility. Mplus output files for these analyses are available at https://osf.io/mkqut/.

### Changes in Personality After Entering Higher Education

Tables 1 to 3 show the means and standard deviations of the Big Five personality traits, locus of control, and risk-taking propensity for the two subsamples at each measurement occasion after propensity score matching on parental and participants’ background characteristics, as well as personality at T1. Changes larger than 2 T-scores are shown in bold, indicating a small effect. The largest changes are obsereved in conscientiousness; in both samples increases exceed 4 T-scores. For all personality traits, the averages at T1 are close to 50, which means that they are similar to the larger youth sample.

**Figure 2:**
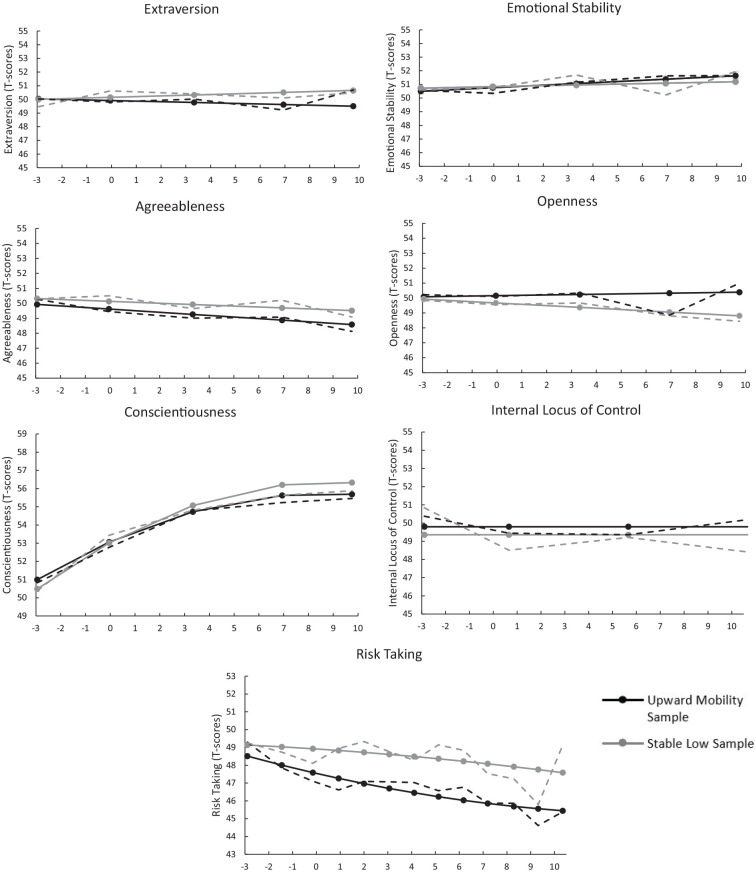
Changes in Personality in the Upward Mobility and Stable Low Sample *Note.* Samples are matched on background variables and personality traits measured at T1. For an overview of all matching variables, see Tables S1–S3. The *x*-axis shows time in years, centered around the transition to higher education (0). Solid lines represent parametric estimates from the latent growth curve models, while dotted lines represent the non-parametric estimates.

We used unconditional growth curve models in both subsamples to test the direction and degree of mean-level change. Tables S5, S6, and Figure 2 present the results of the best-fitting multiple-group linear latent growth curve models. None of the personality traits exhibited significant differences in linear or quadratic change between the upward mobility and stable low subsample across the three time points (Table S7).

Because non-significant slopes might accumulate into significant differences between the groups over time, we tested whether the samples differed in personality at the final time point. We accomplished this by recentering the intercept of the latent growth model at the final time point and testing for differences between the two samples. Only one out of the seven differences was significant, indicating that the stable low sample scored higher on risk-taking propensity compared to the upward mobility sample 10 years after the transition to higher education. However, this difference was small (a difference of 2 T-scores, Wald = 5.30, *p* = .021). All other differences were non-significant. Mplus output files for these analyses are available at https://osf.io/mkqut/.

We also explored differences in personality changes between individuals from the upward mobility sample and the stable high sample. For risk-taking, the stable high sample showed a slightly steeper decline than the upward mobility sample (Figure S1, Table S8). These findings underscore the association between parental and personal educational level and risk-taking propensity. In conjunction with Figure 2, we observed that participants in the stable high sample showed the most substantial decrease, followed by those in the upward mobility sample, while individuals in the stable low sample exhibited the smallest decrease. At the final time point (i.e., 10 years after the transition to university), these differential change patterns resulted in a noticeable gap in risk-taking levels, with the stable low sample scoring over 5 T-scores higher than the stable high sample. However, changes in other personality traits over the 13-year study period did not lead to significant differences in trait levels across the samples.

## Discussion

The present preregistered study investigated whether a cultural shift in the social class environment during the transition to university influenced first-generation students’ Big Five traits, locus of control, and risk-taking propensity. We compared the personality traits of first-generation students to those of young adults with similarly low SES backgrounds who did not transition to university. The results revealed that the two groups already differed in risk-taking propensity at age 17, prior to the university transition, suggesting the presence of selection effects. Specifically, individuals more likely to pursue higher education than their parents tended to be more risk-averse. However, after applying propensity score matching based on participants’ parental and personal background variables (e.g., parental occupational prestige, parental country of origin, and presence of siblings) and personality traits measured at the first assessment, we found little overall evidence that upward educational mobility led to changes in personality traits.

Both samples did show personality change during this life phase, especially in conscientiousness, but these changes did not significantly differ. Given our rigorous case–control design, this suggests that changes in conscientiousness attributed to university attendance may be better explained by genetic or other pre-existing factors, or by other life transitions occurring in both groups (Zisman & Ganzach, 2021). However, we did find a small difference in risk-taking 10 years after the transition to higher education. Specifically, when matching the two samples on risk-taking propensity at the first measurement occasion, small differences in change accumulated, resulting in lower scores on risk-taking scores in the upward mobility sample compared to the stable low sample. The negative link between risk-taking and upward mobility can be explained by both selection and socialization: people who are risk-averse are not only more likely to move up in educational level compared to their parents, but they also become more risk-averse after moving up in social class. A similar trend was observed in continuing-generation students (i.e., the stable high sample), who exhibited an even steeper decline in risk-taking propensity than first-generation students. The steeper decline in risk-taking propensity for participants undergoing educational mobility may indicate a socialization process occurring at university (through social norms or peer influence). This suggests that socialization at university predominantly affects more malleable aspects of personality, as no significant differences in change were found for the other personality traits between the two samples.

Notably, the psychological literature on social class typically identifies a positive relationship between social class and risk-taking, rather than a negative one (Amir et al., 2020). This discrepancy may stem from the frequent use of economic games as proxies for risk-taking, which may not accurately reflect real-life risk behaviors. In contrast, our findings align with studies that examine concrete risk-taking behaviors—such as health-related behaviors and gambling—which often report an inverse relationship between education level and risk-taking propensity in adolescents and young adults (Ahmad et al., 2018; de Winter et al., 2016; Meader et al., 2016). Our results may be more consistent with these studies because our measure—a general self-report scale for risk-taking—may better capture real-life risk-taking behaviors.

Overall, these findings are consistent with Kassenboehmer and colleagues’ (2018) study showing no substantial effects of university education on personality as well as with previous meta-analytic results demonstrating only minor effects of life events on Big Five personality trait changes (Bühler et al., 2023). However, our results surprisingly differ from other lines of research. For instance, research on personality change in the work domain has found stronger effects (e.g., increases in conscientiousness) in response to graduation and the first job (Bühler et al., 2023; Lüdtke et al., 2011).

Our study also found little evidence supporting the selection effects of personality on upward educational mobility, as personality traits did not strongly predict university attendance among our low-SES students. This finding is partly in line with results from Damian and colleagues (2015), who reported that the interaction effects between parental SES and personality are small and not always robust when predicting educational attainment.

The results are also interesting to consider in light of the models of psychology of social class (Manstead, 2018). It appears that the distinct socialization pressures associated with going to university do not significantly influence the relatively less malleable personality traits of first-generation students, at least not in the specific 10-year timeframe of the study. Perhaps, at university, first-generation students self-select into groups with other first-generation students to alleviate social discomfort. It may be that multiple transitions associated with a higher social class-such as acquiring a higher social class peer network or the transitioning into the labor market are necessary to affect one’s personality.

Despite the strengths of using nationally representative longitudinal data and advanced methods like propensity score matching, the findings must be viewed in light of several limitations. First, propensity score matching provides unbiased estimates only if all relevant covariates are included (Rosenbaum & Rubin, 1983), and unmeasured confounders cannot be entirely ruled out. Second, our findings are limited to the German setting and might not be generalizable to countries where tertiary education is less accessible for students with a low-SES background. Finally, we did not account for possible differences in university climates and/or fields of study. Universities and fields of study differ substantially in their level of socio-economic cultures (Browman & Destin, 2016; Dambrun et al., 2009). Perhaps only elite social class environments exert changes in first-generation students’ personalities.

In sum, by following first-generation students before and after their transition to university and comparing their personality development to a control sample similar on parental educational level, parental occupation, and other background characteristics, we believe this paper provided one of the strongest tests to date of whether moving up in educational level compared to your parents leads to personality trait change. Our results showed that while entering university may be a challenging experience for first-generation students and affects their risk preferences, it does not lead to substantial personality trait change.

## Supplemental Material

sj-docx-1-spp-10.1177_19485506251326333 – Supplemental material for Social Class and Personality: The Effects of Educational Mobility on Personality Trait Change Supplemental material, sj-docx-1-spp-10.1177_19485506251326333 for Social Class and Personality: The Effects of Educational Mobility on Personality Trait Change by Anatolia Batruch and Manon A. van Scheppingen in Social Psychological and Personality Science
