# Supplementary material for: Social Class and Personality: The Effects of Educational Mobility on Personality Trait Change
Source: Soc Psychol Personal Sci. 2025 Mar 31;17(2):217–28. doi: 10.1177/19485506251326333 (PMC12795332; doi:10.1177/19485506251326333)
Supplement: sj-docx-1-spp-10.1177_19485506251326333 – Supplemental material for Social Class and Personality: The Effects of Educational Mobility on Personality Trait Change [file sj-docx-1-spp-10.1177_19485506251326333.docx]

***Supplemental Material***

**Social Class and Personality:**

**The Effects of Educational Mobility on Personality Trait Change**

**Propensity Score Matching**

Because propensity score matching does not allow for missing data, we used Bayesian Stochastic regression imputation, which takes into account prediction error and parameter uncertainty in estimating the regression coefficients of the imputation model. Missing data indicators were included in the propensity score model to make sure the treatment and control sample were matched on missing data patterns. The combination of Bayesian Stochastic regression imputation and the inclusion of missing data indicators is found to be an effective approach for dealing with missing data in a propensity score matching context (Coffman et al., 2020; Stuart, 2010).

For each person from the upward mobility sample, the matching model used a nearest neighbor algorithm to find the three best matches based on their propensity scores (Thoemmes & Kim, 2011). To ensure close matches, we utilized a tolerance level on the maximum propensity score distance between matches using a caliper width of .2 standard deviations of the logit of the propensity score (Austin, 2011). We used matching with replacement, which means that respondents in the control sample were allowed to be included more than once. This approach ensured that each participant in the mobility sample could be matched to the nearest control, even if this control was already included in a previous match. Compared to matching without replacement, this approach reduces the risk of matching participants in the mobility sample to controls that are quite different in their propensity scores (Dehejia, 2002).

Table S1 to 3 show the standardized differences on the matching variables between the upward mobility and stable low samples before and after matching. The matching procedure was done separately for the Big Five traits, locus of control, and risk-taking. In general, propensity score matching worked well: standardized differences between the upward mobility and stable low samples decreased and became less than .10. When including all matching variables, there was a standardized difference larger than .10 in locus of control after matching. We therefore chose to exclude some of the variables that contained information about missing data patterns for income, region, owning a house, occupational prestige mother, and year of birth of respondent. These variables showed very small differences between the samples before matching (i.e., Cohen’s *d* varied between .000 and .005) and were less important to control for than locus of control. After removing these variables, propensity score matching lead to good matches on all the variables (i.e., Cohen’s *d* < .10).

| **Table S1.** | | | | | | | |
| --- | --- | --- | --- | --- | --- | --- | --- |
| *Means of Covariates Measured Before the Transition to University in the Upward Mobility Sample and the Stable Low Sample Before and After Matching for the Big Five Personality Traits* | | | | | | | |
| Sample | Upward Mobility | Unmatched Control | Cohen’s  *d* | Matched Control 1 | Cohen’s  *d* | Matched Control 2 | Cohen’s  *d* |
|  | (n = 354) | (n = 1,866) |  | (n = 566) |  | (n = 551) |  |
| Covariate | *M* | *M* |  | *M* |  | *M* |  |
| Propensity score | 0.34 | 0.12 | .93 | 0.33 | .00 | 0.33 | .00 |
| Occupational Prestige Father | 58.68 | 48.53 | .43 | 59.17 | -.03 | 59.22 | -.05 |
| Occupational Prestige Mother | 57.53 | 53.54 | .19 | 56.09 | .07 | 56.34 | .05 |
| Siblings (1 = yes) | 1.12 | 1.11 | .04 | 1.10 | .04 | 1.11 | .02 |
| Income^2^ | 22206.70 | 18472.10 | .24 | 21630.26 | .03 | 21914.52 | .01 |
| Region (1 = West Germany) | 0.84 | 0.78 | .16 | 0.83 | .01 | 0.86 | -.07 |
| Birth Year Father | 1961.75 | 1964.22 | -.39 | 1961.46 | .05 | 1961.93 | -.01 |
| Birth Year Mother | 1964.44 | 1967.20 | -.48 | 1964.30 | .03 | 1964.54 | .00 |
| Country of Origin Father (1 = Germany) | 0.82 | 0.80 | .06 | 0.79 | .08 | 0.83 | -.04 |
| Country of Origin Mother (1 = Germany) | 0.80 | 0.82 | -.06 | 0.80 | .00 | 0.80 | .02 |
| Location of Childhood (1 = medium or large city) | 0.44 | 0.38 | .12 | 0.43 | .02 | 0.39 | .10 |
| Owning home (1 = homeowner) | 0.61 | 0.45 | .34 | 0.57 | .08 | 0.63 | -.06 |
| Gender (1 = Male; 2 = Female) | 1.53 | 1.49 | .06 | 1.55 | -.05 | 1.53 | .00 |
| Age at T1 | 17.18 | 17.13 | .11 | 17.16 | .04 | 17.16 | .04 |
| Birth Year Respondent | 1992.34 | 1994.65 | -.61 | 1992.41 | -.01 | 1992.66 | -.07 |
| Survey Year T1 | 2009.52 | 2011.78 | -.64 | 2009.57 | -.01 | 2009.82 | -.07 |
| Study Participation Length (in Years) | 7.51 | 4.16 | 1.04 | 7.48 | -.01 | 7.18 | .06 |
| Missing Data Occupational Prestige Father | 0.06 | 0.09 | -.16 | 0.05 | .03 | 0.06 | .01 |
| Missing Data Occupational Prestige Mother | 0.11 | 0.11 | -.01 | 0.09 | .06 | 0.12 | -.03 |
| Missing Data Sibling | 0.01 | 0.00 | .07 | 0.00 | .03 | 0.00 | .01 |
| Missing Data Income | 0.00 | 0.00 | -.04 | 0.00 | .00 | 0.00 | .00 |
| Missing Data Region | 0.00 | 0.00 | -.04 | 0.00 | .00 | 0.00 | .00 |
| Missing Data Birth Year Father | 0.03 | 0.03 | -.01 | 0.03 | -.01 | 0.03 | .02 |
| Missing Data Birth Year Mother | 0.00 | 0.00 | .02 | 0.00 | .02 | 0.00 | .00 |
| Missing Data Country of Origin Father | 0.01 | 0.02 | -.03 | 0.02 | -.02 | 0.01 | .07 |
| Missing Data Coutnry of Origin Mother | 0.00 | 0.00 | -.05 | 0.00 | .00 | 0.00 | .00 |
| Missing Data Location of Childhood | 0.01 | 0.00 | .06 | 0.01 | .00 | 0.01 | .02 |
| Missing Data Owning Home | 0.00 | 0.00 | -.04 | 0.00 | .00 | 0.00 | .00 |
| Openness T1 | 50.16 | 48.93 | .14 | - | - | 49.84 | .04 |
| Extraversion T1 | 49.92 | 49.94 | .00 | - | - | 49.46 | .06 |
| Conscientiousness T1 | 50.80 | 50.29 | .05 | - | - | 50.39 | .05 |
| Agreeableness T1 | 50.19 | 49.64 | .06 | - | - | 50.30 | .00 |
| Emotional Stability T1 | 50.53 | 49.66 | .09 | - | - | 50.59 | .00 |
| *Note*. Differences in Cohen’s *d* larger than .1 are shown in bold.  ^2^The logged equivalized and inflation-adjusted annual post-government household income after taxes and transfers. | | | | | | | |
| **Table S2.** | | | | | | | |
| *Means of Covariates Measured Before the Transition to University in the Upward Mobility Sample and the Stable Low Sample Before and After Matching for Locus of Control* | | | | | | | |
| Sample | Upward Mobility | Unmatched Control | Cohen’s  *d* | Matched Control 1 | Cohen’s  *d* | Matched Control 2 | Cohen’s  *d* |
|  | (n = 311) | (n = 1,715) |  | (n = 502) |  | (n = 489) |  |
| Covariate | *M* | *M* |  | *M* |  | *M* |  |
| Propensity score | 0.34 | 0.12 | .94 | 0.33 | .00 | 0.34 | .00 |
| Occupational Prestige Father | 58.16 | 49.40 | .40 | 57.53 | .01 | 57.76 | .01 |
| Occupational Prestige Mother | 58.76 | 53.80 | .24 | 58.46 | .00 | 57.23 | .07 |
| Siblings (1 = yes) | 1.12 | 1.11 | .04 | 1.12 | -.02 | 1.14 | -.06 |
| Income^2^ | 22807.04 | 18898.69 | .23 | 22322.44 | .03 | 21250.07 | .09 |
| Region (1 = West Germany) | 0.80 | 0.77 | .07 | 0.82 | -.05 | 0.78 | .05 |
| Birth Year Father | 1961.58 | 1964.42 | -.47 | 1961.62 | .00 | 1961.71 | -.02 |
| Birth Year Mother | 1964.13 | 1967.36 | -.56 | 1964.05 | .02 | 1964.13 | .00 |
| Country of Origin Father (1 = Germany) | 0.84 | 0.81 | .08 | 0.82 | .04 | 0.82 | .05 |
| Country of Origin Mother (1 = Germany) | 0.80 | 0.83 | -.07 | 0.77 | .08 | 0.78 | .06 |
| Location of Childhood (1 = medium or large city) | 0.45 | 0.38 | .15 | 0.46 | -.02 | 0.45 | .01 |
| Owning home (1 = homeowner) | 0.63 | 0.46 | .36 | 0.62 | .03 | 0.61 | .04 |
| Gender (1 = Male; 2 = Female) | 1.54 | 1.51 | .08 | 1.58 | -.08 | 1.56 | -.04 |
| Age at T1 | 17.18 | 17.11 | .15 | 17.18 | .00 | 17.16 | .04 |
| Birth Year Respondent | 1992.05 | 1995.04 | -.80 | 1992.15 | -.02 | 1992.08 | .00 |
| Survey Year T1 | 2009.24 | 2012.15 | -.84 | 2009.33 | -.02 | 2009.24 | .00 |
| Study Participation Length (in Years) | 8.16 | 4.52 | 1.04 | 7.94 | .04 | 8.15 | .00 |
| Missing Data Occupational Prestige Father | 0.06 | 0.10 | -.18 | 0.07 | -.05 | 0.06 | .00 |
| Missing Data Occupational Prestige Mother | 0.11 | 0.11 | .00 | - | - | - | - |
| Missing Data Sibling | 0.00 | 0.06 | -.94 | 0.00 | .04 | 0.00 | .06 |
| Missing Data Income | 0.00 | 0.00 | .00 | - | - | - | - |
| Missing Data Region | 0.00 | 0.00 | .00 | - | - | - | - |
| Missing Data Birth Year Father | 0.04 | 0.05 | -.06 | 0.05 | -.06 | 0.03 | .02 |
| Missing Data Birth Year Mother | 0.00 | 0.00 | .01 | - | - | - | - |
| Missing Data Country of Origin Father | 0.02 | 0.03 | -.08 | 0.02 | -.02 | 0.02 | -.02 |
| Missing Data Coutnry of Origin Mother | 0.00 | 0.00 | -.05 | 0.00 | .00 | 0.00 | .00 |
| Missing Data Location of Childhood | 0.01 | 0.06 | -.51 | 0.01 | .04 | 0.01 | .01 |
| Missing Data Owning Home | 0.00 | 0.00 | .00 | - | - | - | - |
| Locus of Control T1 | 50.38 | 49.21 | .14 | - | - | 50.84 | -.06 |
| *Note*. Differences in Cohen’s *d* larger than .1 are shown in bold.  ^2^The logged equivalized and inflation-adjusted annual post-government household income after taxes and transfers.  When including all matching variables we initially ended up with a large difference between samples on locus of control (Cohen’s *d* > .1). To improve the quality of the match we removed some of the covariates related to the missing data pattern from the matching procedure. We only removed covariates that showed very small differences (Cohen’s *d* <.01) between the upward mobility and stable low sample. | | | | | | | |

| **Table S3.** | | | | | | | |
| --- | --- | --- | --- | --- | --- | --- | --- |
| *Means of Covariates Measured Before the Transition to University in the Upward Mobility Sample and the Stable Low Sample Before and After Matching for Risk Taking* | | | | | | | |
| Sample | Upward Mobility | Unmatched Control | Cohen’s  *d* | Matched Control 1 | Cohen’s  *d* | Matched Control 2 | Cohen’s  *d* |
|  | (n = 436) | (n = 2,519) |  | (n = 723) |  | (n = 718) |  |
| Covariate | *M* | *M* |  | *M* |  | *M* |  |
| Propensity score | 0.31 | 0.12 | .85 | 0.30 | .00 | 0.30 | .00 |
| Occupational Prestige Father | 58.20 | 49.15 | .37 | 57.08 | .01 | 56.73 | .03 |
| Occupational Prestige Mother | 58.50 | 53.46 | .24 | 58.45 | .00 | 56.57 | .07 |
| Siblings (1 = yes) | 1.11 | 1.11 | .01 | 1.09 | .04 | 1.10 | .02 |
| Income^2^ | 22489.35 | 18629.97 | .26 | 22776.96 | -.02 | 21891.66 | .04 |
| Region (1 = West Germany) | 0.82 | 0.78 | .11 | 0.83 | -.02 | 0.82 | .01 |
| Birth Year Father | 1961.29 | 1963.84 | -.40 | 1961.52 | -.02 | 1961.39 | .01 |
| Birth Year Mother | 1963.93 | 1966.75 | -.49 | 1964.12 | -.01 | 1963.99 | .02 |
| Country of Origin Father (1 = Germany) | 0.81 | 0.79 | .05 | 0.78 | .07 | 0.78 | .08 |
| Country of Origin Mother (1 = Germany) | 0.81 | 0.82 | -.03 | 0.79 | .05 | 0.78 | .06 |
| Location of Childhood (1 = medium or large city) | 0.45 | 0.38 | .13 | 0.44 | .00 | 0.42 | .05 |
| Owning home (1 = homeowner) | 0.62 | 0.45 | .36 | 0.61 | .02 | 0.61 | .01 |
| Gender (1 = Male; 2 = Female) | 1.53 | 1.50 | .07 | 1.56 | -.05 | 1.58 | -.09 |
| Age at T1 | 17.28 | 17.20 | .13 | 17.28 | -.04 | 17.28 | -.02 |
| Birth Year Respondent | 1991.76 | 1994.23 | -.61 | 1991.85 | .00 | 1991.76 | .02 |
| Survey Year T1 | 2009.03 | 2011.43 | -.65 | 2009.13 | -.01 | 2009.04 | .02 |
| Study Participation Length (in Years) | 8.06 | 4.55 | .99 | 7.88 | .02 | 8.03 | -.02 |
| Missing Data Occupational Prestige Father | 0.05 | 0.10 | -.26 | 0.04 | .03 | 0.05 | .00 |
| Missing Data Occupational Prestige Mother | 0.09 | 0.10 | -.04 | 0.09 | .02 | 0.10 | -.02 |
| Missing Data Sibling | 0.01 | 0.04 | -.27 | 0.01 | .05 | 0.01 | .03 |
| Missing Data Income | 0.00 | 0.00 | -.04 | 0.00 | .00 | 0.00 | .00 |
| Missing Data Region | 0.00 | 0.00 | -.04 | 0.00 | .00 | 0.00 | .00 |
| Missing Data Birth Year Father | 0.03 | 0.04 | -.04 | 0.04 | -.02 | 0.03 | .02 |
| Missing Data Birth Year Mother | 0.00 | 0.00 | -.03 | 0.00 | .00 | 0.00 | .00 |
| Missing Data Country of Origin Father | 0.01 | 0.03 | -.13 | 0.01 | .07 | 0.02 | -.03 |
| Missing Data Coutnry of Origin Mother | 0.00 | 0.00 | -.06 | 0.00 | .00 | 0.00 | .00 |
| Missing Data Location of Childhood | 0.01 | 0.04 | -.35 | 0.01 | .04 | 0.01 | .02 |
| Missing Data Owning Home | 0.00 | 0.00 | -.04 | 0.00 | .00 | 0.00 | .00 |
| Risk Taking T1 | 49.26 | 50.67 | -.16 | - | - | 49.25 | .01 |
| *Note*. Differences in Cohen’s *d* larger than .1 are shown in bold.  ^2^The logged equivalized and inflation-adjusted annual post-government household income after taxes and transfers. | | | | | | | |

| **Table S4**  *Logistic Regression of Personality Predicting Educational Mobility Before and After Propensity Score Matching* | | | | |
| --- | --- | --- | --- | --- |
| Predictor |  | Odds Ratio | 95% CI | *p* |
| Extraversion | Before Matching | 1.000 | [0.989, 1.011] | .970 |
|  | After Matching | 0.987 | [0.971, 1.003] | .108 |
| Agreeableness | Before Matching | 1.006 | [0.995, 1.017] | .318 |
|  | After Matching | 0.989 | [0.974, 1.004] | .159 |
| Conscientiousness | Before Matching | 1.005 | [0.994, 1.017] | .369 |
|  | After Matching | 1.004 | [0.988, 1.019] | .648 |
| Emotional Stability | Before Matching | 1.009 | [0.997, 1.022] | .144 |
|  | After Matching | 1.008 | [0.993, 1.024] | .311 |
| Openness | Before Matching | 1.013 | [1.002, 1.024] | .019 |
|  | After Matching | 1.013 | [0.997, 1.030] | .104 |
| Locus of Control | Before Matching | 1.015 | [1.002, 1.028] | .025 |
|  | After Matching | 1.007 | [0.988, 1.027] | .457 |
| Risk Taking | Before Matching | 0.984 | [0.973, 0.994] | .002 |
|  | After Matching | 0.984 | [0.969, 0.999] | .042 |
| *Note.* Predictors were measured at T1, before the age of 20. Upward educational mobility was defined by entering higher education in future waves. Propensity score matching was done on background variables but not on personality traits. For an overview of all matching variables see Table S1-S3. | | | | |

**Latent Growth Curve Models of Personality Traits**

Based on BIC values, the linear growth model with freed variance around the intercept and slope had the best fit (i.e., the lowest BIC value) for openness, extraversion, agreeableness, and emotional stability. For conscientiousness, a quadratic growth curve model with fixed variance around the quadratic slope had the best fit. For locus of control, an intercept-only model with freely estimated variance around the intercept fit best. For risk-taking, a model including linear and quadratic change with freely estimated variance around all growth parameters had the best fit (Table S5, best-fitting models are shown in bold).

| **Table S5** |  |  |  |  |  |  |  |  |
| --- | --- | --- | --- | --- | --- | --- | --- | --- |
| *Fit Indices for Intercept-Only, Linear and Quadratic Models* | | | | | | | |  |
|  |  | Extra-version | Agreeable-ness | Conscien-tiousness | Emotional Stability | Openness | Locus of Control | Risk Taking |
| Model |  | BIC | BIC | BIC | BIC | BIC | BIC | BIC |
| Fixed Intercept-only | | 22065.74 | 22276.94 | 21775.59 | 22455.71 | 22109.02 | 16540.93 | 64947.48 |
| Freed Intercept-only | | 20966.54 | 21778.64 | 21117.93 | 21815.46 | 21432.06 | **16294.87** | 61996.02 |
| Fixed Linear Slope | | 20978.72 | 21788.60 | 20920.73 | 21828.33 | 21443.15 | 16297.45 | 61938.56 |
| Freed Linear Slope | | **20916.50** | **21769.03** | 20907.83 | **21750.68** | **21385.00** | 16322.54 | 61845.76 |
| Fixed Quadratic Slope | | 20929.73 | 21780.92 | **20884.24** | 21762.76 | 21398.57 | 16328.93 | 61860.89 |
| Freed Quadratic Slope | | 20959.05 | 21814.79 | 20913.92 | 21767.66 | 21418.31 | 16362.83 | **61822.59** |
| *Note.* BIC = Bayesian information criterion, lower values indicate better model fit. Lowest value is indicated in bold. We started with the most restricted model (i.e., an intercept-only model with fixed variance) and then test whether adding change parameters and freeing the variance around these parameters improved model fit. | | | | | | | | |

| **Table S6** | | | | | | | | | | |
| --- | --- | --- | --- | --- | --- | --- | --- | --- | --- | --- |
| *Parameter Estimates of Latent Growth Models for the Upward Mobility and Stable Low Subsamples after Propensity Score Matching* | | | | | | | | | | |
| Trait | Subsample | Parameter | *B* | *S.E.* | *p* | 95% CI | *Var* | *S.E.* | *p* | 95% CI |
| Extraversion | Upward Mobility | Intercept | 49.96 | 0.44 | .000 | [49.11, 50.82] | 53.46 | 4.82 | .000 | [44.02, 62.91] |
|  |  | Linear Slope | -0.04 | 0.07 | .517 | [-0.17, 0.09] | 0.52 | 0.15 | .000 | [0.24, 0.81] |
|  | Stable Low | Intercept | 50.10 | 0.49 | .000 | [49.14, 51.06] | 55.72 | 6.12 | .000 | [43.72, 67.72] |
|  |  | Linear Slope | 0.05 | 0.07 | .447 | [-0.08, 0.19] | 0.35 | 0.15 | .020 | [0.05, 0.64] |
| Agreeable-ness | Upward Mobility | Intercept | 49.72 | 0.44 | .000 | [49.46, 50.93] | 49.15 | 4.75 | .000 | [39.83, 58.47] |
|  |  | Linear Slope | -0.11 | 0.07 | .117 | [-0.21, 0.09] | 0.23 | 0.11 | .029 | [0.02, 0.44] |
|  | Stable Low | Intercept | 50.19 | 0.37 | .000 | [49.46, 50.93] | 30.22 | 4.69 | .000 | [39.83, 58.47] |
|  |  | Linear Slope | -0.06 | 0.08 | .414 | [-0.21, 0.09] | 0.41 | 0.18 | .023 | [0.02, 0.44] |
| Conscien-tiousness | Upward Mobility | Intercept | 52.45 | 0.43 | .000 | [51.61, 53.29] | 45.93 | 4.81 | .000 | [36.51, 55.35] |
|  |  | Linear Slope | 0.68 | 0.09 | .000 | [0.50, 0.86] | 0.34 | 0.13 | .008 | [0.09, 0.59] |
|  |  | Quadr.Slope | -0.04 | 0.01 | .001 | [-0.06, -0.01] | - | - | - | - |
|  | Stable Low | Intercept | 52.26 | 0.40 | .000 | [51.48, 53.04] | 35.99 | 3.26 | .000 | [29.61, 42.38] |
|  |  | Linear Slope | 0.83 | 0.10 | .000 | [0.63, 1.01] | 0.20 | 0.09 | .030 | [0.02, 0.37] |
|  |  | Quadr.Slope | -0.04 | 0.01 | .000 | [-0.06, -0.02] | - | - | - | - |
| Emotional Stability | Upward Mobility | Intercept | 50.68 | 0.46 | .000 | [49.77, 51.59] | 55.23 | 5.54 | .000 | [44.37, 51.59] |
|  |  | Linear Slope | 0.09 | 0.07 | .213 | [-0.05, 0.23] | 0.40 | 0.13 | .003 | [0.14, 0.23] |
|  | Stable Low | Intercept | 50.80 | 0.42 | .000 | [49.98, 51.61] | 44.22 | 4.09 | .000 | [36.21, 52.23] |
|  |  | Linear Slope | 0.04 | 0.08 | .642 | [-0.12, 0.19] | 0.69 | 0.17 | .000 | [0.35, 1.02] |
| Openness | Upward Mobility | Intercept | 50.13 | 0.39 | .000 | [49.37, 50.90] | 34.37 | 4.08 | .000 | [26.38, 42.37] |
|  |  | Linear Slope | 0.02 | 0.07 | .721 | [-0.11, 0.16] | 0.35 | 0.13 | .006 | [0.10, 0.60] |
|  | Stable Low | Intercept | 49.76 | 0.47 | .000 | [48.84, 50.67] | 52.83 | 6.60 | .000 | [39.90, 65.75] |
|  |  | Linear Slope | -0.09 | 0.07 | .225 | [-0.23, 0.05] | 0.50 | 0.22 | .021 | [0.08, 0.93] |
| Locus of Control | Upward Mobility | Intercept | 49.80 | 0.37 | .000 | [49.07, 50.52] | 26.46 | 3.36 | .000 | [19.86, 33.05] |
|  | Stable Low | Intercept | 49.36 | 0.37 | .000 | [48.63, 50.08] | 22.78 | 3.79 | .000 | [15.35, 30.21] |
| Risk Taking | Upward Mobility | Intercept | 47.86 | 0.33 | .000 | [47.20, 48.51] | 37.14 | 2.99 | .000 | [31.27, 43.01] |
|  |  | Linear Slope | -0.33 | 0.07 | .000 | [-0.47, -0.18] | 0.51 | 0.21 | .014 | [0.10, 0.91] |
|  |  | Quadr.Slope | 0.01 | 0.01 | .254 | [-0.01, 0.03] | 0.00 | 0.00 | .365 | [0.00, 0.01] |
|  | Stable Low | Intercept | 49.00 | 0.33 | .000 | [48.34, 49.65] | 35.72 | 3.03 | .000 | [29.78, 41.66] |
|  |  | Linear Slope | -0.08 | 0.09 | .382 | [-0.26, 0.10] | 1.33 | 0.24 | .000 | [0.86, 1.80] |
|  |  | Quadr.Slope | 0.00 | 0.01 | .659 | [-0.02, 0.01] | 0.01 | 0.00 | .000 | [0.00, 0.01] |
| *Note.* Samples are matched on background variables and personality traits measured at T1. For an overview of all matching variables see Table S1-S3. For the upward mobility sample, the intercept was centered at the year before entering higher education. For the stable low sample, the intercept was centered at age 19, which is the average age one year before entering higher education in our sample. | | | | | | | | | | |

| **Table S7.**  *Tests of Differences in Growth Parameters Between Stable Low Sample and Upward Mobility Sample After Propensity Score Matching.* | | | | | |
| --- | --- | --- | --- | --- | --- |
|  |  | Test of Differences in Change | | Test of Differences in Intercept (centered at last time point) | |
| Personality Trait | Model | Wald | *p* | Wald | *p* |
| Extraversion | Intercept + Linear Slope | 1.00 | .318 | 1.19 | .275 |
| Agreeableness | Intercept + Linear Slope | 0.18 | .669 | 0.69 | .406 |
| Conscientiousness | Intercept + Linear + Quadratic Slope | 1.64 | .441 | 0.32 | .572 |
| Emotional Stability | Intercept + Linear Slope | 0.23 | .629 | 0.13 | .721 |
| Openness | Intercept + Linear Slope | 1.28 | .258 | 2.07 | .150 |
| Locus of Control | - | - | - | 0.72 | .397 |
| Risk Taking | Intercept + Linear + Quadratic Slope | 5.13 | .077 | 5.30 | .021 |
| *Note.* Samples are matched on personality traits at the initial measurement occasion. For the models including quadratic change the Wald test provides a combined test of differences between the sample s in both the linear and quadratic slope. | | | | | |

| **Table S8.**  *Tests of Differences in Growth Parameters Between Stable High Sample and Upward Mobility Sample* | | | | | | | |
| --- | --- | --- | --- | --- | --- | --- | --- |
|  |  |  | | Test of Differences in Intercept | | | |
|  |  | Test of Differences in Change | | Centered at First Time Point | | Centered at Last Time Point | |
| Personality Trait | Change Parameters | Wald | *p* | Wald | *p* | Wald | *p* |
| Extraversion | Linear Slope | 1.83 | .176 | 0.00 | .987 | 1.59 | .207 |
| Agreeableness | Linear Slope | 0.62 | .431 | 1.40 | .237 | 1.94 | .163 |
| Conscientiousness | Linear + Quadratic Slope | 1.85 | .396 | 2.69 | .101 | 0.02 | .886 |
| Emotional Stability | Linear Slope | 0.64 | .422 | 1.90 | .168 | 0.00 | .973 |
| Openness | Linear Slope | 11.17 | .001 | 4.02 | .045 | 4.36 | .037 |
| Locus of Control | Linear Slope | 3.41 | .065 | 5.63 | .018 | 0.23 | .636 |
| Risk Taking | Linear + Quadratic Slope | 6.30 | .043 | 0.35 | .556 | 6.35 | .012 |

*Note.* The x-axis shows time in years, centered around the transition to higher education (0). Solid lines represent parametric estimates from the latent growth curve models, dotted lines represent the non-parametric estimates.

**Figure S1**

*Changes in Personality in the Upward Mobility and Stable High Sample*
